# Brief Strategic Therapy for Bulimia Nervosa and Binge Eating Disorder: A Clinical and Research Protocol

**DOI:** 10.3389/fpsyg.2019.00373

**Published:** 2019-03-08

**Authors:** Giada Pietrabissa, Gianluca Castelnuovo, Jeffrey B. Jackson, Alessandro Rossi, Gian Mauro Manzoni, Padraic Gibson

**Affiliations:** ^1^Psychology Research Laboratory, Istituto Auxologico Italiano IRCCS, Milan, Italy; ^2^Department of Psychology, Catholic University of the Sacred Heart, Milan, Italy; ^3^Department of Human Development and Family Science, Virginia Tech, Falls Church, VA, United States; ^4^Department of Philosophy, Sociology, Education, and Applied Psychology, Section of Applied Psychology, University of Padua, Padua, Italy; ^5^Faculty of Psychology, eCampus University, Novedrate, Italy; ^6^Bateson Clinic, Dublin, Ireland; ^7^Hesed House, Dublin, Ireland

**Keywords:** brief strategic therapy, bulimia nervosa, binge eating disorder, longitudinal study, psychotherapy process and outcome

## Abstract

**Background:** Although cognitive behavioral therapy is the gold standard treatments for bulimia nervosa (BN) and binge eating disorder (BED), evidence for its long-term efficacy is weak. Empirical research support the efficacy of brief strategic therapy (BST) in treating BN and BED symptoms, but its statistical significance still need to be investigated.

**Objective:** To statistically test the long-term efficacy of the BST treatment protocols for BN and BED through one-year post-treatment.

**Methods:** A two-group longitudinal study will be conducted. Participants will be sequentially recruited for inclusion in a state-funded community psychotherapy clinic. Multilevel growth curve modeling will be used to estimate the average growth trajectories from baseline to one year after treatment termination for the severity of the BN and BED features as measured by the Eating Disorder Examination Questionnaire.

**Discussion:** Findings from this study will clarify the impact of BST in treating BN and BED symptoms.

**Conclusion:** Translating research into practice may inform how to ensure high-quality patient care.

## Introduction

At any given point in time 1.0% of young women and 0.1% of young men will suffer from bulimia nervosa (BN) or binge eating disorder (BED) in Europe (Keski-Rahkonen and Mustelin, 2016). BN and BED are psychopathological conditions characterized by abnormal or disturbed eating behavior (American Psychiatric Association, 2013). They are usually comorbid with other psychiatric conditions, including depression, anxiety, and substance abuse disorders (Keski-Rahkonen and Mustelin, 2016), and are associated with substantial economic and social burden (Agh et al., 2016; Erskine et al., 2016; Le et al., 2018).

Bulimia nervosa is characterized by recurrent and distressing binge eating episodes marked by the consumption of a large amount of food in a brief period and feeling out of control while doing so. These episodes are followed by inappropriate compensatory weight loss behaviors, such as laxative use, induced vomiting, fasting, and excessive exercise (American Psychiatric Association, 2013). Individuals with BN are obsessed and preoccupied with their shape and weight from which they derive their self-worth (Spangler and Allen, 2012).

Like those with BN, individuals suffering from BED present episodes of binge eating associated with marked distress, but in absence of inappropriate compensatory weight control methods (American Psychiatric Association, 2013). Individuals with BED remain, therefore, at high risk of developing obesity and its associated risk factors (i.e., hypertension, orthopedic problems, diabetes) (McCuen-Wurst et al., 2018).

Bulimia nervosa and BED treatments can be delivered in a variety of settings and levels of care, and reduction of symptoms is generally considered the first goal of therapy.

Cognitive behavioral therapy (CBT) is the most researched and recommended evidence-based treatment for BN and BED (Grilo, 2017). It aims to modify distorted beliefs about body image and alter the behaviors that are associated with and perpetuate EDs (Fairburn et al., 1993). Despite the widespread support for the efficacy of CBT (Murphy et al., 2010; Agras et al., 2017; Linardon and Brennan, 2017), a recent meta-analysis indicated that results are mixed (Linardon et al., 2017), and a significant proportion of people with BN or BED – particularly those with comorbid personality disorders (Grilo et al., 2007; Sallet et al., 2010; Farstad et al., 2016; Simpson et al., 2018) – have also been found to fail to respond to or not to complete CBT treatment (Waller et al., 2009).

In an effort to improve the quality and generalizability of BN and BED clinical treatment outcomes, the implementation of alternative treatments to CBT in non-research settings, and the evaluation of long-term treatment efficacy through the collection of follow-up data are needed (Tolin et al., 2015). Empirical investigations have shown brief strategic therapy (BST) to be clinically effective in treating several psychiatric disorders, and the results of recent experimental research have been encouraging enough in confirming its statistical efficacy and effectiveness to merit further investigation (Nardone and Portelli, 2005; Castelnuovo et al., 2011; Pietrabissa et al., 2016, 2017; Jackson et al., 2018).

### Brief Strategic Therapy (BST)

The BST approach conceptualizes problems as the product of a complex process of recursive interactions between individuals and their reality, in which individuals’ repeated ineffective attempts to solve the problem inadvertently maintain or exacerbate it (Nardone et al., 1999). People have a natural inclination to repeat solutions that have proven to be functional in the past; however, generalizing such solutions to different situations, or reapplying the same strategies when they no longer produce the desired effects, creates and maintains a maladaptive way of thinking about and reacting to the problem known as a dysfunctional *perceptive-reactive system* (Nardone and Brook Barbieri, 2010).

Unlike CBT, BST does not focus on identifying the causes of a problem, but rather on what maintains and reinforces the problem in the present, simultaneously intervening to disrupt dysfunctional perceptive-reactive systems through the implementation of specific heuristic maneuvers (Nardone and Portelli, 2005). The results of implemented strategies provide important information regarding how the problem is maintained, leading to the development of specific treatment protocols for several mental health disorders (i.e., knowing through changing) (Nardone and Watzlawick, 2005).

In BST, the skilful use of language is crucial for helping patients to follow therapeutic prescriptions: whereas the types of language used in CBT to facilitate explanation tend to be logical-rational and indicative (Fairburn et al., 1993), in BST, therapeutic communication tends to consist of strategic dialog and analogical language (i.e., metaphors, anecdotes, aphorisms, stories) to reframe attempted solutions as threatening and dangerous (i.e., fearing restricting rather than binging), thus subverting the perception of the problem (Nardone and Salvini, 2007). Hypnotic and performative language are also used to create the persuasive effect that helps establish the therapeutic alliance and create motivation to follow prescriptions.

#### Bulimia Nervosa (BN)

Bulimia nervosa is an uncontrollable desire to binge eat linked to a dissolute desire for consumption of food. The primary attempted solution for controlling weight gain consists of severe caloric restriction – in presence or absence of compensatory behaviors (i.e., use of laxative, induced vomiting, fasting, and excessive exercise) – with the paradoxical effect of increasing the urge to binge in a vicious cycle that reinforces the problem (Nardone et al., 1999; Nardone, 2003). Assessment consists of understanding individuals’ *perceptive-reactive system* to differentiate among the following four specific typologies of BN clinical presentation (Nardone et al., 1999).

(1) *Overweight/Obese*: People with this type of BN are generally overweight or obese, as they find food and eating immensely pleasurable, but struggling to consistently stay on a diet. They are usually aware of and have capitulated to their dysfunctional relationship with food. They are also using cellulite to protect themselves from challenging aspects of life. Overweight/obese-BN possess extrinsic motivation to change eating habits for health reasons.
(2) *Overweight/Obese for Relational Protection*: People with this type of BN tend to be emotionally sensitive and their cellulite provides an insulating barrier that defends against relational suffering.
(3) *Weight Cycling*: People with the weight cycling type of BN typically experience weight fluctuation because they go on and off diets in a vicious dietary cycle of alternating control (dieting) with loss of control (binge eating) (Nardone et al., 1999; Nardone, 2003).
(4) *Vomiting*: The presence of the compensatory behavior of vomiting has been found to have a unique perceptive-reactive system characterized by the obsessive search for pleasure and strong sensations. The pleasure is not derived from food (as it is for the other BN types), but from ritualized vomiting, which represents a “functional” attempted solution to lose weight or to avoid gaining weight while continuing to eat (Nardone et al., 1999). When the cycle of eating and vomiting is continuously repeated, it becomes an increasingly enjoyable ritual with a phase of desire to which a phase of consumption follows, ending with a phase of discharge. Intentional vomiting after eating loses the original scope of losing weight and gains the objective of feeling overwhelming sensations of pleasure.

In the initial stage of the first three classes of BST treatment for BN, it is crucial to establish a therapeutic alliance, engage the patients, and bypass resistance to change (Nardone and Brook Barbieri, 2010). Individuals with BN are, in fact, collaborative in their intentions, but are also hindered by the fact that they rely on their strength of will. Depending on the patients’ perceptive-reactive system – the therapist may either use the *miracle fantasy* technique (i.e., What would need to happen in your life for you to be able to say, “My problem has been resolved?”) – to induce a form of positive self-deception by building a sort of prophecy that enables clients to form a strong image of a possible life without the problem – or ask the patients to think how to voluntarily worst their situation (*how worst* technique). This results in the recognition that the behaviors designed to protect them from binging are instead high-risk maintaining factors.

Further, family members are prescribed the *conspiracy of silence* (i.e., avoid talking about or acting on the problem) to interrupt attempted solutions who may be unwittingly colluding with keeping the current eating system intact (Nardone, 2003).

In the second stage of BST treatment for BN, the primary intervention is the *paradoxical diet* in which the individual is asked to think of what they would like to eat the most and carefully prepare and eat as much of those things as they want during mealtimes. The paradoxical logic underpinning the intervention is that “the only way to get rid of temptation is to yield to it” (Wilde, 1890), thus reducing the frequency of eating in favor of the pleasure of eating, and indirectly diminishing the daily amount of food the individual consumes (Nardone and Brook Barbieri, 2010). The paradoxical diet is usually accompanied by the *if you do once you do five* paradoxical prescription (i.e., From now until the next time we see each other, every time you eat something out of your three daily meals, you will have to eat it no more and no less than five times). It is aimed at transforming the guilty pleasure of eating outside of mealtimes into torture provoking extinction (Nardone et al., 1999). The concomitant usage of these two paradoxical prescriptions usually leads to a complete unblocking of the bulimic symptomatology in a few sessions.

In the third stage of the therapy, patients are paradoxically encouraged to regularly engage in *small transgressions* in their eating rules to further ameliorate their eating patterns (i.e., Each day treat yourself to a small portion of food that you crave but that you tell yourself you should not have). In fact, “if you allow it, you can do without it; if you don’t allow it, it will become irresistible.” The fourth stage of BST treatment for BN is focused on helping individuals assume increased responsibility over their positive changes.

Moving to the vomiting subtype of BN, the first phase of the BST treatment has the main objective of making the person realize his/her actions to be pleasure-driven, by presenting the eating and vomiting sequence as a metaphorical *meeting with a “secret lover.”*

Other than the *conspiracy of silence*, to block the attempted solutions of the family system the *list of foods to vomit* may be also prescribed. This strategy foresees a family member to ask the patient “What would you like to eat and vomit today?” and to display the food with a note – “Things to eat and vomit for [the patient’s name]” – in public. This task (a) completely subverts the attempted solution of the family system of hiding food to prevent the patient from binging, at the same time (b) ruining the transgressive connotation of the ritual, therefore (c) functioning as a strong inhibiting factor.

In the second phase of the therapy the *interval technique* is prescribed to deprives the ritual of its intrinsic enjoyment. The pleasurable sequence of exciting fantasy, consummation and then discharge is altered by the insertion of a time interval (which progressively increase during treatment – making the vomiting more and more difficult and unpleasant) between the binging and the vomiting. If the patient won’t accept the above prescription, s/he will be progressively guided in increasing the search for pleasure by building the *perfect blowout.*

Once stop vomiting – since afraid of gaining weight – patients also normalize their relationship with food, and the *paradoxical diet* in then prescribed (third phase).

The fourth stage and final stage of BST treatment for vomiting aims at increasing individuals’ self-confidence and responsibility over their achievements (Nardone and Brook Barbieri, 2010).

#### Binge Eating Disorder (BED)

Similarly to BN, individuals with BED alternate between control over ingested food and the inevitable subsequent loss of control, but the vicious cycle occur more frequently. Also, the BST conceptualization of BED differs from that of BN in that individuals with BED engage in the compensatory practices of fasting. The postponement of eating through fasting tends to magnify the desire for food, typically resulting in the eventual surrender to the pleasure of binging (Nardone and Brook Barbieri, 2010; Castelnuovo et al., 2011). Primary interventions in BST treatment for BED consists of a *fear of fasting* reframe aimed at helping patients realize that even if fasting might seem the best way to lose weight, abstaining from eating sets up the next loss of control over food and binging leads to gaining even more weight (Nardone and Brook Barbieri, 2010). As individuals perceive that fasting leads to binging, they usually start eating more regularly without having been asked directly to do so; as Pascal stated, “people are generally better persuaded by the reasons which they have themselves discovered than by those which have come into the mind of others” (Pascal et al., 1623–1662).

Direct and paradoxical maneuvers are then prescribed over the course of treatment for improving individuals’ relationship with food by adopting more balanced, flexible, and healthy eating behaviors. The fourth stage focuses on increased understanding of how the therapeutic process was effective and on enhancing self-esteem and self-confidence. Notably, whereas the *paradoxical diet* is considered one of the initial interventions for the treatment of BN, this prescription represents the goal of the therapy with individuals suffering from BED and is prescribed in the final stages of the treatment (Nardone et al., 1999; Table 1).

**Table 1:** Comparison of bulimia nervosa (BN) and binge eating disorders (BED) features and treatment stages according to the brief strategic therapy (BST) approach.

	**BULIMIA NERVOSA**				
	**Overweight/obese** arriving in therapy for health problems	**Overweight/obese** for affective-relational protection	**Weight Cycling**	**Vomiting**	**BINGE EATING DISORDER**

*** Attempted Solutions***	Control (diet)			Compensatory behaviors – vomiting	Control (fasting or quanti-qualitative over-controlled diet)

*** Perceptive Reactive System***			Obsessive-compulsive disorder based on pleasure		

*** Intervention Logic***				Paradox	

**TREATMENT STAGES**					

**I**	• How worst OR Miracle fantasy • Conspiracy of silence			• Meeting with a “secret lover” • Conspiracy of silence • List of foods to vomit	• “Fear of fasting” restructuring • Conspiracy of silence.
					
**II**	• Paradoxical diet AND If you do once you do five			• Interval technique OR • Perfect blowout	

**III**	Small food transgression(s) during well-balanced nutritious diet			Paradoxical diet	

**IV**	Redefinition of a new balance, support self-efficacy and conclusion of the therapy				


## Study Aim

The present study is designed to test the long-term efficacy of the BST treatment for BN and BED by assessing eating disorder symptomatology in a non-research outpatient clinic through one-year post-treatment.

## Methods

### Study Design

A non-randomized observational design will be used to determine if the BST protocols for BN and BED are associated with statistical improvement from baseline assessment through one-year post-treatment. Despite the presence of a control group would allow for greater reliability of research findings, the present represents the only treatment option. Still, assessment of outcomes under observational studies informs the extent to which an intervention does what is intended to do in routine circumstances – thus allowing the optimization of healthcare interventions in ecological settings.

The study was approved by the research ethics board of Hesed House. Psychotherapy service registered charity number: CHY 1678.

### Participants

Participants diagnosed with BN and BED who self-referred to a state community psychotherapy clinic in Dublin, Ireland will be consecutively screened for inclusion into the study. The inclusion criteria will consist of the following: (1) 18 to 65 years old, (2) a diagnosis of BN or BED according to the DSM-5 (American Psychiatric Association, 2013), and (3) providing written informed consent to participate in the study. Exclusion criteria for the participants from the study were: (1) visual impairment that makes it difficult to complete written questionnaires, (2) hearing or cognitive impairment that makes it difficult to receive psychotherapy, and (3) presentation of comorbid psychiatric disorders, including substance abuse (Keski-Rahkonen and Mustelin, 2016), that could bias the results of the study. Eligible patients will be invited to participate in the study and provided with detailed information about the research aims and procedures. Those who agree to participate and sign the informed consent form will be included in the study.

### Outcome Measure

The *Eating Disorder Examination Questionnaire 6.0* (EDE-Q 6.0) (Fairburn et al., 2008) will be used as a measure of eating disorder feature severity. The EDE-Q 6.0 is a 28-item self-report questionnaire based on the EDE interview (Fairburn and Beglin, 1994). The items assess the frequency of behaviors indicative of an eating disorder over a 28-day period. The test is scored on a 7-point scale from 0 to 6 and has four subscales (i.e., restraint; eating concern; shape concern and weight concern) as well as a global score. The EDE-Q 6.0 has generally demonstrated adequately reliability and validity for eating-related pathology and specific disordered eating behaviors (Mond et al., 2004).

### Procedure

The study was approved by the research ethics committee of the clinic. Screening for inclusion will be conducted for consecutive patients at admission to the clinic by a clinical psychologist and psychotherapist who will be involved in the study screening and data collection stages only. Patients who will be eligible to participate will be subsequently informed about the study and invited to take part in the study. After completing the pre-treatment assessment (baseline), patients will receive 45–50 min of BST face-to-face treatment every 2 weeks for BN or BED based on their diagnosis for an anticipated total of 8 to 10 sessions. Participants will be assessed using the outcome measure (EDE-Q) at pre-treatment (T1), post-treatment (T2; approximately 16–20 weeks after the pre-treatment assessment), 6-month follow-up (T3), and 1-year follow-up (T4). During the treatment stage (T1 and T2), assessment will be conducted in a dedicated room at the clinic, whereas follow-up assessment (T3 and T4) will be conducted online.

### Psychotherapists and Treatment Fidelity

All psychotherapy sessions will be provided by two licensed brief strategic therapists with substantial experience in the treatment of BN and BED. To ensure treatment fidelity, therapy sessions will be video-recorded and randomly audited by two senior licensed BST psychotherapists. Only patients who agree to be video registered during the psychotherapy sessions will be included in the study.

### Sample Size

The minimum sample size required to conduct this study was computed by using an *a priori sample size calculator* (G^∗^Power 3.1.9.2 software (Faul et al., 2007, 2009) for Fisher *f*’s tests. Participants – both BN than BED – will be measured with the EDE-Q 6.0 (Fairburn et al., 2008) at four time points: (1) at admission to the clinic, (2) at discharge, (3) at 6-month follow-up, and (4) at one year follow-up. *Clinical diagnosis* (BN *vs.* BED) was classified as between-group variable, and *time* was classified as within-subject variable. The *a priori* statistics (partial η^2^ and correlation between measures) were derived by considering previous longitudinal studies using the EDE-Q 6.0 (Fairburn et al., 2008) as measure of the range and severity of eating disorders’ features. The *a priori* partial η^2^ was set to assume a value of 0.020 (Fairburn et al., 2009) – small effect size (Cohen, 1988; Eid et al., 2017) – that provides a Cohen’s *f* equal to 0.143. Moreover, the Type I error (α) rate was set at 0.05 (two-sided) and the Power (1 – β) was set at 0.80, according to general guidelines (Cohen, 1988). In line with previous studies, the *a priori* correlation between repeated measures was set at 0.60 (Bardone-Cone and Boyd, 2007; Rose et al., 2013). Finally, sphericity was assumed. Results showed that there is an almost 81% chance of correctly rejecting the null hypothesis of no significant effect of the interaction with 28 subjects per group for a total of 56 participants.

### Statistical Analysis

Data analyses will be performed using the R software (R Core Team, 2014), and lme4 package (Bates et al., 2015). First, preliminary analyses will be performed to test the assumptions of parametric statistics: if (strong) violations are detected, robust methods or data transformation will be applied. The EDE-Q 6.0 will be examined with the intention-to treat (ITT) analyses approach, with missing data replaced by baseline observations carried forward (BOCF) or last observation carried forward (LOCF), assuming no improvement for non-responders, as reported in previous studies (Castelnuovo et al., 2011). Dropouts will be excluded from the study.

The independent samples *t*-test will be used to examine between-group differences (BN *vs*. BED) in the EDE-Q 6.0 variables at all time-points, with attention to changes in the scores. The chi-square statistic will be used to test the association between treatment groups and socio-demographic variables (gender, age, marital status and employment status), and correlation analysis will be used to test the association between quantitative variables. Finally, multilevel growth curve model analysis will be used to estimate the average growth trajectories from baseline to one year after treatment for the EDE-Q 6.0 scores with corrected effect sizes (Hedges’ *g*) and 95% confidence interval.

## Discussion

This paper provides a description of a proposal to evaluate the long-term efficacy of BST for the treatment of BN and BED in a real-world setting. Whilst the superiority of BST over CBT for the long-term treatment of BED has been shown in a previous study (Jackson et al., 2018), no research has yet investigated the long-term efficacy of BST for the treatment of BN. It is expected that findings will increase knowledge of the impact of BST for BED, while – for the first time – statistically testing the long-term effects of the BST protocol for BN. Due to its novelty, no hypothesis was made about the possible contribution of BST for the treatment of BN. Instead, it has been hypothesized that BST will promote sustained remission of BED symptoms.

Despite a few limitations – including the absence of a control group and the use of a single psychological measure – the research design is adapted to the clinical setting with the aim to move research into practice. Therefore, research findings will lack of generalizability, but will be useful to generate new formal hypotheses and to develop more precise research design in real-world settings.

## Author Contributions

PG conceived the study. GP, AR, and JBJ wrote the manuscript. GMM and GC supervised the work.

## Conflict of Interest Statement

The authors declare that the research was conducted in the absence of any commercial or financial relationships that could be construed as a potential conflict of interest.
